# Comparison of Long-Term Outcomes and Refractive Stability following SMILE versus SMILE Combined with Accelerated Cross-Linking (SMILE XTRA) 

**DOI:** 10.1155/2022/4319785

**Published:** 2022-02-28

**Authors:** Sheetal Brar, Skanda Sriganesh, Smith Snehal Sute, Sri Ganesh

**Affiliations:** Nethradhama Super Speciality Eye Hospital, Bangalore, India

## Abstract

**Purpose:**

To compare the long-term safety, efficacy, predictability, and refractive stability following SMILE versus SMILE combined with accelerated cross-linking (SMILE XTRA), and to specifically study the regression patterns following the two procedures.

**Methods:**

This retrospective study included 54 eyes of SMILE and 54 eyes of SMILE XTRA treated for normal and borderline cases of myopia/myopic astigmatism, respectively, based on certain predefined topographic features and risk factors. Patients in both the groups were matched for age and refractive error. The mean postoperative follow-up for the SMILE group was 22.18 ± 10.41 months and the SMILE XTRA group was 21.81 ± 10.19 months.

**Results:**

At the end of follow-up, the mean sphere, cylinder, and SE reduced to −0.03, −0.09, and −0.08 D in the SMILE group and −0.06, −0.15, and −0.13 D in the SMILE XTRA group. 96% and 93% eyes remained within ±0.50 D in SMILE and SMILE XTRA groups, respectively, and 94% eyes maintained an UDVA of 20/20 or better in the SMILE as well as SMILE XTRA groups. Safety and efficacy indices for the SMILE group were 1.03 and 1.00. For the SMILE XTRA group, the safety and efficacy indices were 1.00 and 0.99. No eye in either group had postoperative ectasia or enhancement performed for significant residual refractive error.

**Conclusion:**

Both the SMILE and SMILE XTRA groups exhibited comparable visual outcomes, safety, and efficacy. Contrary to the belief, combination of prophylactic CXL with SMILE did not result in a hyperopic shift in the long term. No eye in either group encountered postoperative ectasia; however, further follow-up is suggested to establish the long-term effects on refractive and corneal stability following SMILE XTRA, as all the eyes treated in this group were borderline.

## 1. Introduction

Despite the potential advantages of SMILE over femtosecond laser-assisted in situ keratomileusis (FS-LASIK) and photorefractive keratectomy (PRK), the procedure is characterized by a steeper learning curve, during which intraoperative complications may occur. Suction loss, black spots, dense opaque bubble layer, lenticule tears, incision tears, and inability to find the lenticule are some of the intraoperative complications of SMILE that were reported earlier. [1–5].

SMILE was proposed to be biomechanically more stable compared to LASIK and PRK [6]. However, ectasia was shown to occur even after SMILE, with most of these cases having borderline or abnormal preoperative topography [7]. Therefore, preoperative evaluation for a corneal refractive surgery has received significant attention in the recent years. Various risk scoring systems and tomographic indices combined with biomechanics have come into existence to help a refractive surgeon identify corneas at risk [8–12]. Along with these advanced screening systems, a new form of refractive surgery, i.e., combined collagen cross-linking (CXL) with the primary corneal refractive surgery has emerged in the recent years, aiming at improving postoperative corneal biomechanical stability, thereby preventing the risk of future keractasia [12–14]. This was based on the proven evidence that CXL lead to halting of progression and corneal stabilization of keratoconic corneas [15–20]. Newer CXL protocols [21–23],including the STARE-X protocol [21]and use of SafeCross® riboflavin solution chemically boosted corneal cross-linking [22], demonstrated effective results in halting keratoconus progression in 2-year follow-up and improving DLD by a factor of 20%, without adverse events for corneal endothelium, respectively. Since there is enough evidence to support that CXL can stabilize keratoconus, it was proposed that prophylactic CXL when combined with various corneal refractive surgeries may prevent the risk of future keractasia in borderline eyes.

This class of refractive surgeries, popularly known as “XTRA procedures,” can be combined with PRK, LASIK, and SMILE and is typically performed in cases where the topographic/tomographic indices or the clinical history is suggestive of “at risk” corneas.

However, many refractive surgeons are reserved to combine corneal refractive procedures with cross-linking due to reasons such as potential risk of haze, overcorrection, or hyperopic outcome due to progressive flattening as a result of cross-linking, additional cost, and lack of knowledge and experience, etc. However, evidence is growing that a refractive surgery with simultaneous cross-linking is safe and effective in preventing ectasia without any significant side effects [24–28]. Especially, when combined with SMILE, it was shown to be beneficial in preventing ectasia when used to treat borderline corneas [29]. However, there is a paucity of long-term data on the efficacy and stability of SMILE XTRA when compared to SMILE. The present retrospective study was thus conducted with the aim of comparing the long-term safety, efficacy, predictability, and refractive stability following SMILE versus SMILE XTRA and to specifically study the regression patterns following the two procedures.

## 2. Methods

The present retrospective study was approved by institutional ethics committee of Nethradhama Super Speciality Eye Hospital, Bangalore, and adhered to the tenets of Declaration of Helsinki. Data were collected from electronic medical records of all patients who had refractive surgery performed for correction of myopia or myopic astigmatism with SMILE or SMILE XTRA procedure from January 2017 to December 2018. Only those patients who had a minimum follow-up of 12 months were included in the study.

Preoperative evaluation was performed using the combined corneal tomography (OCULUS Pentacam® HR, Wetzlar, Germany) and biomechanics (Corvis ST, Oculus). Based on the tomographic and biomechanical evaluation and patient's age, refractive error, and additional risk factors, eyes were categorized into “normal” or “borderline” based on the following criteria [27]:

1. Corneal thickness <480 microns
2. Residual bed thickness between 250 and 280 microns
3. Refractive Error >−6.00 D spherical equivalent (SE)
4. Pentacam criteria: Belin Ambrosio display final *D*-value >1.65
5. Corvis-ST criteria: Corvis Biomechanical Index (CBI) >0.5 and Tomographic Biomechanical Index (TBI) >0.29
6. Additional risk factors = age <30 years, family history of keratoconus, or history of eye rubbing

If none of the above criteria were present, eyes were classified as “normal”, whereas if 3 or more of the above criteria were present, eyes were classified as “borderline” for SMILE surgery. All eyes in the “normal” group underwent a routine SMILE procedure, whereas in the “borderline” group, some eyes underwent SMILE XTRA and some eyes underwent only SMILE. The decision regarding the procedure in the “borderline” eyes was influenced by factors such as additional cost, surgeon's intuition, and patient's willingness. Patients who did not undergo SMILE XTRA due to any reason were strictly advised against eye rubbing and were called for 6-monthly follow-ups. They were also asked to report earlier if they noticed any drop/change in their vision. Only eyes with a minimum follow-up of 12 months were included in the study.

### 2.1. Surgical Procedure

During treatment planning, a similar nomogram (10% overcorrection) was used for both the SMILE and SMILE XTRA groups.

As regards the surgical procedures, all procedures were performed by 2 experienced SMILE surgeons (SG and SB). SMILE was performed with the VisuMax FS laser (Carl Zeiss Meditec, Jena, Germany) using the following parameters: a cap thickness of 100–120 microns, an optical zone of 6-7 mm, energy cut index between 28 and 32 (140–160 nJ), and a superior access incision of 2-3 mm.

For SMILE XTRA, the surgical steps were as follows: (1) SMILE performed following the standard protocol (described above); (2) 0.22% riboflavin in saline (Vibex XTRA, Avedro, Waltham, MA) or 0.23% riboflavin (Peschke L, Huenenberg, Switzerland) injected into the interface and allowed to diffuse for 60 s, followed by irrigation of the interface with balanced salt solution; and (3) UV-A irradiation through the cap using a power of 45 mW/cm^2^ for 75 s, delivering a total energy 3.4 J/cm^2^.

Postoperative medication regimen consisted of topical 0.3% ofloxacin (Exocin®, Allergan, Irvine, U.S.A.) 4 times/day for 3 days after SMILE and 7 days after SMILE XTRA, 0.1% prednisolone acetate eye drops (Pred Forte®, Allergan, Irvine, U.S.A.) 4 times/day for 4 weeks (tapering weekly), and lubricants 4 times/day for 4 weeks or more following both procedures.

### 2.2. Statistical Analysis

Microsoft excel statistical tool pack was used to analyze the data and perform the statistical analysis. Data were checked for normality before subjecting to statistical tests. Based on the results of normality tests, parametric or nonparametric tests were applied. Intergroup comparisons were performed using the independent *t*-tests and intragroup comparisons were performed using the paired *t*-tests. A *p* value of 0.05 or less was considered statistically significant.

## 3. Results

A total of 108 eyes from 54 patients (*n* = 27 patients in the SMILE group, and *n* = 27 patients in the SMILE XTRA group) undergoing a bilateral refractive surgery for myopia or myopic astigmatism correction were included in study. Both groups were comparable with respect to preoperative age, sphere, cylinder, SE, and corneal astigmatism; however, the SMILE XTRA group had a significantly thinner central pachymetry and steeper keratometry (both K1 and K2) (Table 1). Regarding the intraoperative treatment parameters, there was no significant difference between the two groups in terms of actual refraction treated (after application of a 10% nomogram), cap thickness, optical zone, and residual bed thickness. However, the maximum and minimum lenticule thickness (LT) values were significantly lower for the SMILE XTRA group (LT, max = 87.48 ± 21.70 *μ*, min = 13.65 ± 6.42 *μ*) compared to the SMILE group (LT, max = 96.53 ± 22.90 *μ*, min = 16.85 ± 8.25 *μ*) (Table 1).

The mean follow-up in the SMILE group was 22.18 ± 10.41 (range 12–54) months and in the SMILE XTRA group was 21.81 ± 9.19 (range 12–52) months, *p*=0.45.

### 3.1. Visual and Refractive Results

**Figure 1:**
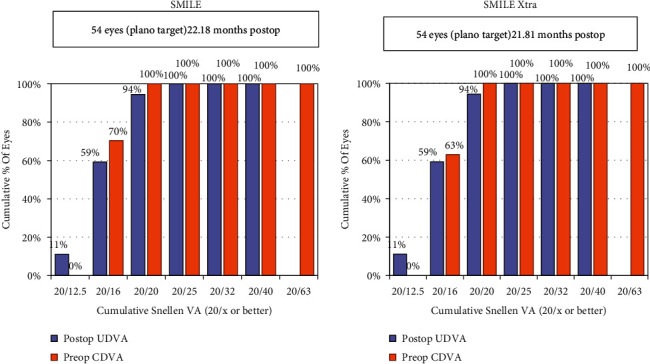
Cumulative histogram for binocular uncorrected distance visual acuity (UDVA).

At the end of the mean follow-up, the % age of eyes seeing 20/20 or better was 94% (*n* = 51) in the SMILE group as well as in the SMILE XTRA group (Figure 1).

No significant difference in the mean postoperative UDVA was observed between both the groups (*p*=0.56) (Table 2).

The efficacy index (postoperative UDVA/preoperative CDVA) was 1.00 and 0.99 for the SMILE and SMILE XTRA groups, respectively.

**Figure 2:**
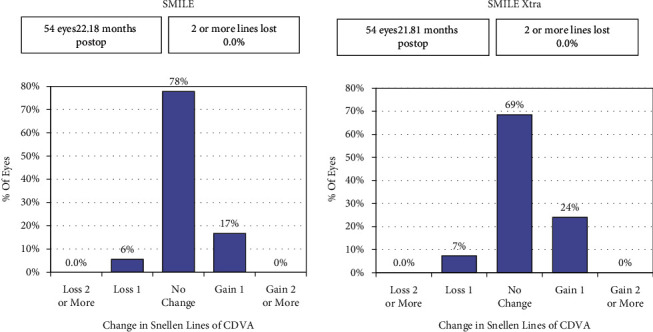
Histogram showing the change in the Snellen lines of corrected distance visual acuity (CDVA).

As regards the safety, 95% eyes (*n* = 51) in the SMILE group had postoperative CDVA same or better, compared to 93% (*n* = 50) in the SMILE XTRA group. No eye in either group had loss of 2 lines or more (Figure 2).

The safety index (postoperative CDVA/preoperative CDVA) was 1.03 and 1.00 for the SMILE and SMILE XTRA groups, respectively.

**Figure 3:**
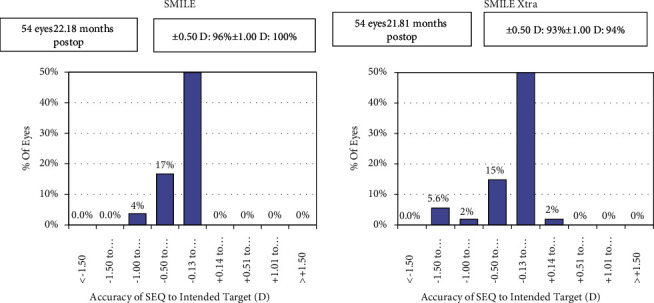
Histogram showing the accuracy to the intended spherical equivalent refraction.

**Figure 4:**
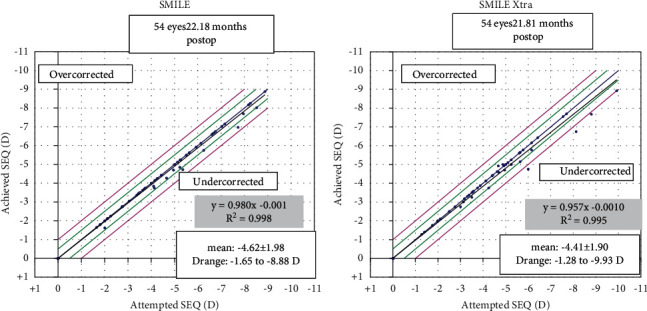
Spherical equivalent refraction predictability.

Ninety-six percent (*n* = 52) eyes in the SMILE group and 93% (*n* = 50) eyes in the SMILE XTRA group had postoperative SE predictability between ±0.5 D. All eyes in the SMILE group were within ±1.00 D, whereas all eyes in the SMILE XTRA group were within ±1.50 D (Figures 3 and 4).

The mean residual SE at the end of the mean follow-up was −0.08 ± 0.18 D in the SMILE group versus −0.13 ± 0.3 D in the SMILE XTRA group; however, the difference was not statistically significant (*p*=0.34) (Table 2).

**Figure 5:**
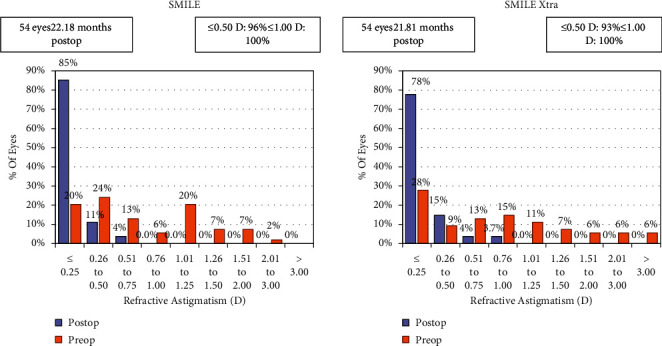
Histogram showing change in refractive astigmatism.

In terms of cylinder correction, 96% eyes (*n* = 52) in the SMILE group versus 93% eyes (*n* = 50) in the SMILE XTRA group were within ±0.50 D, and all eyes in both the groups were within ±1.00 D of cylinder correction (Figure 5).

## 4. Stability

**Figure 6:**
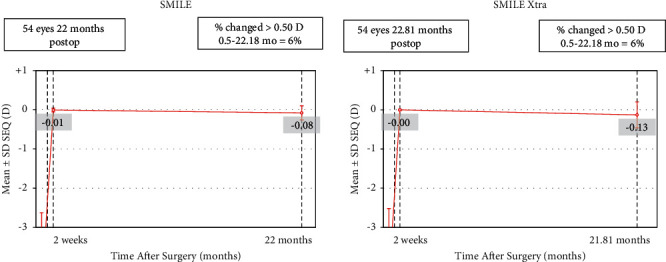
Stability of spherical equivalent refraction.

In the SMILE group, the mean postoperative SE at 2 weeks was −0.006 ± 0.05 D which increased to −0.08 ± 0.18 D at the mean follow-up. On the other hand, in the SMILE XTRA group, the mean SE increased from −0.002 ± 0.01 D at 2 weeks to −0.13 ± 0.33 D at the end of the mean follow-up (Figure 6). The change in SE in both the groups compared to 2 weeks was found to be statistically significant (Tables 3 and 4).

Mean regression in the SMILE group was 0.08 D, which was less, compared to the SMILE XTRA group (0.13 D), the difference between the two groups, however, was not statistically significant (*p*=0.34).

## 5. Long-Term Complications

All eyes in the SMILE group had a clear interface, while 4 eyes in the SMILE XTRA group had evidence of mild interface haze (grade 0-1) at the last follow-up. However, no patient complained of any visual disturbances due to this. When asked about the spectacle independence and quality of vision through a subjective questionnaire, the mean score of overall satisfaction (out of 100) was 98.2% in the SMILE group, and 95.4% in the SMILE XTRA group (Table 5).

## 6. Discussion

Inspired by the reports of safety and efficacy of LASIK XTRA, in 2015, we explored SMILE XTRA as a potential treatment option for borderline cases [29]. However, our initial cases of SMILE XTRA were reserved for selected cases of keratoconus suspect corneas. It may be argued that why SMILE XTRA was not performed in the borderline eyes, other than those who were keratoconus suspects, as the primary procedure when ectasia was anticipated. Multiple reasons influenced our decision making. First, there were some cases where topography was slightly borderline, but corneal thickness and residual bed thickness were relatively good. Considering the perceived biomechanical advantage of SMILE (no vertical cut), over LASIK; no flap-related complications and with proper counseling, one may be tempted to treat such cases. Other reasons were increased cost and theoretical risk of haze development, due to which, this option we reserved only for eyes which were indeed at risk of ectasia.

The current literature on SMILE XTRA suggests that combined SMILE and prophylactic accelerated cross-linking does not affect the safety and efficacy of the procedure [30]. In 2015, we published the first outcomes of SMILE XTRA in a prospective case series of 40 eyes of 20 myopic patients with moderate to high risk of ectasia (Randleman Scoring ≥3). The safety and efficacy indices observed in our study were 1.29 and 1.04 at the end of 1 year. CDVA remained stable and no complications such as keratitis, ectasia, regression, or endothelial decompensation were observed [29]. Two eyes that developed Grade 2 corneal haze, resolved within 3 months following treatment with topical steroids. It may be noteworthy to mention that the mild CXL related anterior stromal haze that accompanies the procedure is not visually significant and does not lead to reduction in CDVA. As seen from our data, the safety profile of both SMILE and SMILE XTRA was similar in both groups with no eye losing more than 1 line of CDVA in either group. Osman et al. [28] in their retrospective comparison study observed a similar efficacy index in both the SMILE XTRA group (1.09) and SMILE group (1.12) at 2-year follow-up, suggesting that CXL did not have a significant impact on the uncorrected visual acuity when combined with SMILE. They also observed a high safety index of 1.29 with SMILE XTRA in their study. Besides the above studies on borderline corneas, a study by Graue-Hernandez et al. evaluated the safety and efficacy of SMILE XTRA on 15 forme fruste keratoconus eyes. Their results suggested that SMILE combined with accelerated cross-linking was safe and effective in stabilizing these eyes over a follow-up ranging from 12 to 24 months [31].

The protocol of SMILE XTRA used in our series appears to be effective for preventing ectasia in susceptible eyes, as all eyes remained stable by the end of the follow-up. Recent studies, however, report using different riboflavin concentration, soak time, UV-A irradiation power, and duration to perform combined SMILE and accelerated cross-linking However, none of the eyes which underwent SMILE XTRA in the previously published studies progressed to ectasia. The present study, with a follow-up ranging from 1 to 4 years in both the groups, provides a substantial anecdotal evidence regarding the potentially enhanced stability provided by the simultaneous accelerated CXL, as no eye progressed to ectasia in this series.

Studies have found that collagen cross-linking results in [28, 30, 31] progressive corneal flattening over many years after the procedure [32, 33]. This is one of the main reasons why most refractive surgeons do not prefer simultaneous prophylactic cross linking along with a refractive surgery, as it may potentially lead to a hyperopic result and changes in the refractive outcome. However, we did not observe a significant difference in the mean regression between the two procedures, at almost the same post-op mean follow-up period of 21 months. Even though the same nomogram (10% over correction) was applied to both groups, the mean regression in the SMILE XTRA group was slightly higher compared to the SMILE group (0.13 D vs 0.08 D), although the difference was not significant. This may suggest that the UV protocol used in the study may be just sufficient to prevent ectasia. The cylinder in the SMILE XTRA group at the mean follow-up was higher compared to the SMILE group (although nonstatistically significant), which may suggest that probably we need a longer follow-up to observe these eyes, which may potentially result into ectasia, since they all were borderline eyes to start with. On the other hand, this result may also be interpreted that possibly, it is the accelerated cross-linking which is just holding an ectasia, which may otherwise have become evident by now. The good stability and minimal regression in the SMILE group at the long term, may suggest that SMILE itself may be a stable procedure in a majority of cases and XTRA may only be reserved for suspect cases where the risk of ectasia is higher. This is also evident from the long-term studies recently published on SMILE, wherein a minimal regression of −0.35 ± 0.66 diopters over the 10-year period was observed following SMILE [34].

Nevertheless, our study adds to the existing knowledge on the refractive surgery and simultaneous accelerated cross-linking, especially related to the SMILE XTRA procedure by observing no ectasia, any significant haze or any hyperopic over correction in the borderline eyes treated and followed up for a mean duration of 21 months and longest duration of 4 years. However, since all these eyes were borderline and “at risk” for postoperative ectasia, they definitely call for even longer and closer follow-ups, as the prophylactic CXL may just be delaying the onset of ectasia, which may occur over subsequent course of time. Thus, the long-term safety, efficacy, stability, and effects on corneal stabilization following SMILE XTRA when used to treat borderline corneas, still remain to be established.

## Figures and Tables

**Table 1:** Patient demographics and preoperative data.

Parameter (mean ± SD)	SMILE (*n* = 54)	SMILE XTRA (*n* = 54)	*p* value
Total no. of eyes	54	54	—
Total no. of patients	27	27	—
Male: female	11 : 16	12 : 15	—
Age (years)	25.96 ± 2.71	25.85 ± 4.06	0.90
Sphere (D)	−4.24 ± 1.84	−4.036 ± 1.94	0.56
Cylinder (D)	−0.71 ± 0.68	−0.95 ± 1.06	0.17
SE (D)	−4.61 ± 1.98	−4.41 ± 1.89	0.57
CDVA (logMAR)	−0.06 ± 0.04	−0.06 ± 0.05	0.85
K1 (D)	42.67 ± 1.4	43.68 ± 1.77	≤0.001
K2 (D)	43.68 ± 1.41	44.87 ± 1.54	≤0.001
Astigmatism (D)	1.02 ± 0.55	1.19 ± 0.73	0.19
CCT (*μ*)	527.88 ± 29.47	515.29 ± 26.45	0.02
Thinnest pachymetry (*μ*)	523.83 ± 29.48	510.27 ± 26.84	0.01

VisuMax diagnostic and treatment data			
Sphere (D)	−4.43 ± 1.9	−4.23 ± 2.01	0.58
Cylinder (D)	−0.80 ± 0.68	−0.95 ± 1.1	0.39
Cap thickness (*μ*)	120 ± 0	120 ± 0	1.00
Optical zone (*μ*)	6.36 ± 0.30	6.27 ± 0.29	0.14
Minimum lenticule thickness (*μ*)	16.85 ± 8.25	13.65 ± 6.42	0.02
Maximum lenticule thickness (*μ*)	96.53 ± 22.90	87.48 ± 21.70	0.03
RST (*μ*)	314.13 ± 31.30	303.75 ± 30.65	0.08

UDVA: uncorrected distance visual acuity; D: diopter; SE: spherical equivalent; CDVA: corrected distance visual acuity; K: keratometry; CCT: central corneal thickness; RST: residual stromal thickness.

**Table 2:** Postoperative data at mean follow-up.

Parameter (mean ± SD)	SMILE (*n* = 54)	SMILE XTRA (*n* = 54)	*p* value
Longest follow-up (months)	22.18 ± 10.41	21.81 ± 9.19	0.89
UDVA (logMAR)	−0.05 ± 0.07	−0.06 ± 0.08	0.56
Sphere (D)	−0.037 ± 0.14	−0.060 ± 0.25	0.55
Cylinder (D)	−0.09 ± 0.20	−0.14 ± 0.28	0.28
SE (D)	−0.08 ± 0.18	−0.13 ± 0.33	0.34
CDVA (logMAR)	−0.08 ± 0.06	−0.09 ± 0.05	0.37
K1 (D)	39.63 ± 1.61	40.50 ± 2.03	0.007
K2 (D)	40.44 ± 1.62	41.44 ± 2.02	0.005
Corneal astigmatism (D)	0.82 ± 0.39	0.84 ± 0.40	0.90
CCT (*μ*)	455.33 ± 29.57	442.85 ± 26.65	0.02
Thinnest pachymetry (*μ*)	453.01 ± 29.23	440.35 ± 25.97	0.01

UDVA: uncorrected distance visual acuity; D: diopter; SE: spherical equivalent; CDVA: corrected distance visual acuity; K: keratometry; CCT: central corneal thickness.

**Table 3:** Visual and refractive parameters 2 weeks post-op versus mean follow-up in the SMILE group.

Parameter (mean ± SD)	Post-op 2 weeks	Last follow-up	*p* value
UDVA (logMAR)	−0.07 ± 0.04	−0.05 ± 0.07	0.23
Sphere (D)	−0.00 ± 0.03	−0.037 ± 0.14	0.10
Cylinder (D)	−0.00 ± 0.05	−0.09 ± 0.20	0.002
SE (D)	−0.00 ± 0.05	−0.08 ± 0.18	0.003
CDVA (logMAR)	−0.08 ± 0.05	−0.08 ± 0.06	1.00
K1 (D)	39.58 ± 1.72	39.63 ± 1.61	0.88
K2 (D)	40.36 ± 1.71	40.44 ± 1.62	0.79
Corneal astigmatism (D)	0.77 ± 0.39	0.82 ± 0.39	0.46
CCT (*μ*)	449.31 ± 30.32	455.33 ± 29.57	0.29
Thinnest pachymetry (*μ*)	446.83 ± 29.77	453.01 ± 29.23	0.27

UDVA: uncorrected distance visual acuity; D: diopter; SE: spherical equivalent; CDVA: corrected distance visual acuity; K: keratometry; CCT: central corneal thickness.

**Table 4:** Visual and refractive parameters 2 weeks post-op versus mean follow-up in the SMILE XTRA group.

Parameter (mean ± SD)	Post-op 2 weeks	Last follow-up	*p* value
UDVA (logMAR)	−0.08 ± 0.13	−0.06 ± 0.08	0.24
Sphere (D)	0.00 ± 0.00	−0.062 ± 0.25	0.08
Cylinder (D)	−0.00 ± 0.03	−0.15 ± 0.28	≤0.001
SE (D)	−0.00 ± 0.01	−0.13 ± 0.33	0.005
CDVA (logMAR)	−0.08 ± 0.13	−0.07 ± 0.06	0.63
K1 (D)	40.34 ± 2.00	40.50 ± 2.03	0.67
K2 (D)	41.10 ± 2.06	41.44 ± 2.02	0.38
Corneal astigmatism (D)	0.79 ± 0.34	0.84 ± 0.40	0.49
CCT (*μ*)	435.20 ± 23.96	442.85 ± 26.65	0.11
Thinnest pachymetry (*μ*)	433.87 ± 24.88	440.35 ± 25.97	0.19

UDVA: uncorrected distance visual acuity; D: diopter; SE: spherical equivalent; CDVA: corrected distance visual acuity; K: keratometry; CCT: central corneal thickness.

**Table 5:** Questionnaire and mean scores for evaluation of spectacle independence, patient satisfaction, and quality of vision.

*What is your level of spectacle independence after surgery?*			
(a) Totally dependent on spectacles for all work (0–3.99)	(b) Partially dependent for certain work (4–7.99)	(c) Completely independent of spectacles (8–10)	
SMILE = 9.4			
SMILE XTRA = 9.0			

*Do you experience any dysphotopsia symptoms such as glare or halos? If yes, grade the same as per the following*			
(a) Minimal/nil and occasional (0–3.99)	(b) Moderate and frequent (4–7.99)	(c) Severe and persistent (8–10)	
SMILE = 2.2			
SMILE XTRA = 3.5			
*Do you experience any dry eye symptoms? If yes, grade the same as per the following*			
(a) Minimal/nil and occasional (0–3.99)	(b) Moderate and frequent (4–7.99)	(c) Severe and persistent (8–10)	
SMILE = 2.4			
SMILE XTRA = 3.8			

*How would you grade the quality of your vision after surgery?*			
(a) Poor (0–2.99)	(b) Good (3–4.99)	(c) Very good (5–7.99)	(d) Excellent (8–10)
SMILE = 9.1			
SMILE XTRA = 8.2			

*How would you grade your overall satisfaction after the procedure?*			
(a) Not satisfied at all (0–30.99)	(b) Somehow satisfied (31–60.99)	(c) Moderately satisfied (61–89.99	(d) Fully satisfied (90–100)
SMILE = 98.2%			
SMILE XTRA = 95.4%			

No eye in either group progressed to ectasia or required an enhancement procedure for significant residual refractive error during the course of the study.

## Data Availability

The data can be made available on request from the institutional ethics committee in-charge of Nethradhama Super Speciality Eye Hospital, Bangalore, Dr. Sandhya who can be contacted at sandhyakrish@gmail.com.
